# Interaction Networks in Yeast Define and Enumerate the Signaling Steps of the Vertebrate Aryl Hydrocarbon Receptor

**DOI:** 10.1371/journal.pbio.0020065

**Published:** 2004-03-16

**Authors:** Guang Yao, Mark Craven, Norman Drinkwater, Christopher A Bradfield

**Affiliations:** 1McArdle Laboratory for Cancer Research, University of Wisconsin Medical SchoolMadison, WisconsinUnited States of America; **2**Department of Biostatistics and Medical Informatics, University of Wisconsin Medical SchoolMadison, WisconsinUnited States of America

## Abstract

The aryl hydrocarbon receptor (AHR) is a vertebrate protein that mediates the toxic and adaptive responses to dioxins and related environmental pollutants. In an effort to better understand the details of this signal transduction pathway, we employed the yeast S. cerevisiae as a model system. Through the use of arrayed yeast strains harboring ordered deletions of open reading frames, we determined that 54 out of the 4,507 yeast genes examined significantly influence AHR signal transduction. In an effort to describe the relationship between these modifying genes, we constructed a network map based upon their known protein and genetic interactions. Monte Carlo simulations demonstrated that this network represented a description of AHR signaling that was distinct from those generated by random chance. The network map was then explored with a number of computational and experimental annotations. These analyses revealed that the AHR signaling pathway is defined by at least five distinct signaling steps that are regulated by functional modules of interacting modifiers. These modules can be described as mediating receptor folding, nuclear translocation, transcriptional activation, receptor level, and a previously undescribed nuclear step related to the receptor's Per–Arnt–Sim domain.

## Abstract 2

4500 yeast deletion mutants, transformed with a reporter construct that is sensitive to dioxin, were used to create a protein-interactive map that outlines five distinct steps required for dioxin-mediated cell signaling

## Introduction

The aryl hydrocarbon receptor (AHR) is a ligand-activated transcription factor found in a variety of vertebrate species. The AHR is a prototype member of the Per–Arnt–Sim (PAS) superfamily of signaling molecules. Members of this superfamily regulate cellular responses to a variety of environmental stimuli, including pollutants, hypoxia, and external light cues (Gu et al. 2000). Our initial interest in AHR biology arose from its pivotal role in mediating the adaptive metabolic response to both polycyclic aromatic hydrocarbons (PAHs) and the toxic effects of more potent agonists like the halogenated dioxins (Schmidt and Bradfield 1996; Whitlock 1999). More recently, it has been observed that the AHR plays an important role in normal vascular development, suggesting the existence of an endogenous ligand (Lahvis et al. 2000). From the broader perspective, the AHR can be viewed as a prototype of all PAS protein signaling. That is, what we learn about AHR biology will have a direct influence on how we think about PAS-mediated hypoxia, circadian, and developmental pathways.

An initial understanding of AHR signal transduction has resulted from the biochemical and molecular studies that have been performed over the past two decades (Schmidt and Bradfield 1996; Whitlock 1999). The resultant model holds that the unliganded AHR resides in the cytoplasm, where it is associated with a dimer of the chaperone protein Hsp90 and cochaperones such as ARA9/XAP2 and p23 (Pongratz et al. 1992; Carver and Bradfield 1997; Ma and Whitlock 1997; Meyer et al. 1998; Kazlauskas et al. 1999). Upon binding ligands, the cytoplasmic AHR translocates to the nucleus, where it dimerizes with another PAS protein known as ARNT. The AHR–ARNT heterodimer then binds to specific dioxin-responsive enhancers (DREs) and transactivates a battery of genes encoding xenobiotic-metabolizing enzymes, most notably *CYP1A1*, *CYP1A2*, and *CYP1B1* (Schmidt and Bradfield 1996; Whitlock 1999). Transactivation of target genes has been shown to be mediated through a variety of histone acetyltransferases (HATs) and SWI/SNF coactivators, such as SRC, p300/CBP, and BRG-1 (Kobayashi et al. 1997; Beischlag et al. 2002; Wang and Hankinson 2002).

Although the initial model of AHR signaling provides a valuable framework, its completeness has not yet been assessed. That is, we have no estimates of the total number of gene products involved in AHR signaling, nor can we be sure we have identified all the important steps. Without these estimates, it is difficult to gauge how much or how little we understand about this pathway. In an effort to address these issues, we employed the comprehensive set of gene deletions available in a yeast model system to systematically identify gene products that influence AHR function. We then employed a protein interaction network (PIN) strategy to provide a framework to describe AHR signaling. By coupling both computational and experimental annotations, we were able to deduce the minimum number of genetic loci and signaling events required for AHR signaling.

## Results

### Rationale

A number of laboratories have demonstrated that the yeast Saccharomyces cerevisiae is a valuable model system for the study of signaling by mammalian nuclear receptors (Garabedian and Yamamoto 1992; McEwan 2001). Although there is no yeast ortholog of the AHR, it has been also shown that AHR signaling can be recapitulated in yeast and that this system can be used to identify novel players in AHR biology (Carver et al. 1994; Whitelaw et al. 1995). The experimental advantages of S. cerevisiae as a tool to study AHR signaling are related to the yeast's fundamental similarities with mammalian systems, the more thorough characterization of its smaller genome, and the availability of its specific genomic tools, such as arrayed deletions of each individual open reading frame (ORF) and large-scale databases describing protein and genetic interactions (Winzeler et al. 1999; Resnick and Cox 2000; Kennedy 2002; Mewes et al. 2002; Xenarios et al. 2002). These convenient genomic tools allowed us to employ a systematic approach to identify gene products involved in the AHR pathway and to interpret them in the context of a protein interaction network. Owing to a lack of corresponding reagents/databases, such an approach is not yet feasible for the study of AHR signaling in more complex eukaryotic systems such as human or mouse.

### Identification of AHR Modifiers by a High-Throughput Deletion Array Screen

**Figure 1:**
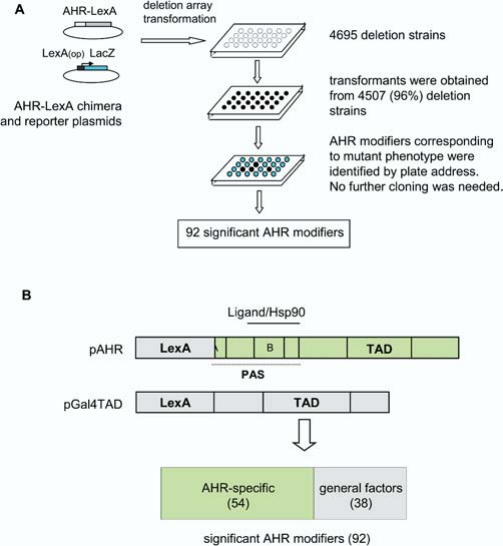
High-Throughput Deletion Array Screen for AHR Modifiers (A) The flow chart of the deletion array screen. Each individual deletion strain was transformed with the AHR–LexA chimera and *LacZ* reporter constructs using a 96-well microtiter plate transformation approach. The AHR-dependent reporter activity of each deletion strain was examined with a 384-well plate-based fluorescence assay method. A total of 92 deletion strains were identified that displayed AHR signaling significantly different from the *wt* control. (B) Identification of “AHR-specific” modifiers. The effect of modifier deletions on the AHR pathway was compared with their effect on a Gal4TAD control pathway. It was found that 54 deletions influenced AHR signaling specifically, whereas 38 deletions corresponded to general factors. See text for details.

In earlier attempts to identify AHR modifiers in yeast, it was demonstrated that genetic screens can be performed more efficiently by using an AHR construct that is fused to the DNA-binding domain of the bacterial LexA protein (AHR–LexA) (Carver et al. 1994; Whitelaw et al. 1995). This chimeric system removes the requirement for ARNT and allows our screens to be more specific for those mutations/modifiers that directly influence AHR function. Using this system, we set out to identify gene products that play important roles in AHR signaling (Figure 1A).

To accomplish this screen, we employed the yeast deletion strains made available by the *Saccharomyces* Genome Deletion Project (Winzeler et al. 1999). We developed a high-throughput approach to efficiently transform each deletion strain with two plasmids, one harboring the AHR–LexA chimera (pCEN-AHR) and the other, a LexA operator-driven LacZ reporter. Of the 4,695 available deletion strains, 4,507 (96%) were successfully transformed with the complete AHR signaling system (i.e., both plasmids). In the primary screen, we selected transformants that exhibited a 4-fold or greater change in AHR response as compared to the wild-type (*wt*) BY4742 strain (*p* < 10^–6^). To minimize false positives, we selected clones that influenced signaling at no less than two of the six concentrations of agonist tested. In addition, we retested each positive strain in a secondary screen with another AHR system containing the same LacZ reporter and a high-copy AHR–LexA chimera (pAHR) (Carver 1996). By these criteria, 92 deletion strains were identified that reproducibly displayed a significant change in AHR signaling as compared to the *wt* strain (Table S1).

To eliminate those deletions that influenced the AHR pathway in a nonspecific manner, each of the 92 deletion strains was examined with a control plasmid pGal4TAD (see Materials and Methods). This construct harbors the transcriptional activation domain (TAD) of Gal4p fused to the LexA DNA-binding domain and was cotransformed into each deletion strain with the LacZ reporter (Figure 1B). Of the 92 deletions, 38 were observed to also influence pGal4TAD signaling. We concluded that these deletions either represented general players in both pathways or exhibited nonspecific effects through their influence on, e.g., the common LexA domain, plasmid maintenance, or cell growth rate. Therefore, the inclusion of the pGal4TAD control led us to eliminate 38 nonspecific factors and identify 54 deletions that appeared to influence the AHR pathway in a specific manner.

Of these “AHR-specific” factors, Hsc82p and Cpr7p were previously described AHR modifiers, and the other 52 were novel (Carver et al. 1994; Whitelaw et al. 1995; Miller 2002) (Table S2). The analysis of the annotated function of these AHR modifiers revealed that they were associated with a great variety of cellular functions (Table S3). For many of these annotations, their direct association with AHR signaling appeared elusive. Therefore, in order to appreciate the function of identified modifiers in the AHR pathway, an information framework was required to put them in context.

### Portrayal of the AHR–PIN

Recent experiments from a number of laboratories have provided data to support the idea that protein interaction network (PIN) can be used to portray the workings of complex biological systems (Schwikowski et al. 2000; Ge et al. 2001; Ideker et al. 2001; Tong et al. 2002). To investigate how identified modifiers and their interactions influence AHR signaling, we constructed a modifier network (AHR–PIN) based on known protein and genetic interactions derived from the DIP and MIPS databases (Mewes et al. 2002; Xenarios et al. 2002). Our AHR–PIN map is comprised of “nodes” and “links.” A “node” is a graphic depiction of a protein or locus, and a “link” is a line between two nodes in the map that depicts the known interaction between them. As yeast protein–protein interactions identified to date are still far from saturating and are heavily biased towards proteins of high abundance, genetic interactions were also included in the network building as a complement (Tong et al. 2002; von Mering et al. 2002). In the AHR–PIN, protein interactions are depicted with black lines, and genetic interactions are labeled in red. In addition, nodes also come in two types, “M-nodes” and “I-nodes.” We refer to the protein or locus that has an identified effect on the AHR pathway as the “M-node,” or modifier node, and refer to the nonmodifier node that is required on a path to connect two M-nodes as the “I-node,” or intervening node.

**Figure 2:**
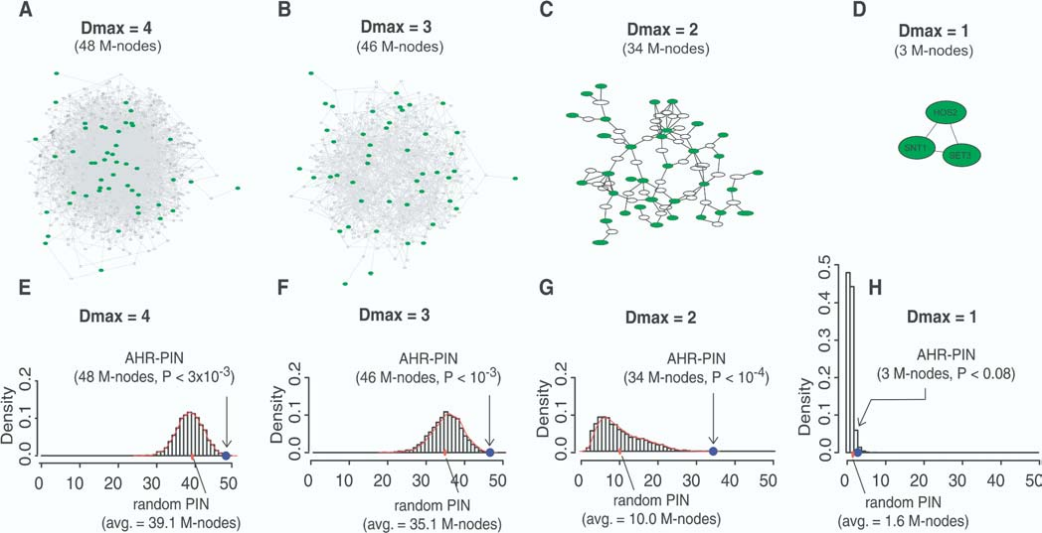
AHR–PIN versus Random PINs (A–D) AHR–PINs at various D_max _levels. AHR modifiers are highlighted with bigger green nodes. A total of 48, 46, 34, and three AHR modifiers are interconnected in the AHR–PINs with D_max_ values of 4, 3, 2, and 1, respectively. (E–H) Distribution of random PINs at various D_max _levels in histogram. Each distribution graph represents 5,000 randomly generated PINs. The density estimation curve (in red) is plotted on top of the histogram where applicable. The number of M-nodes in the AHR–PIN and the average number of M-nodes in random networks are marked in each distribution graph. See text for details.

In an effort to determine the most informative PIN, we examined how the structure and complexity of the map was influenced by the choice of the maximally allowed number of links between any two M-nodes (we refer to this value as D_max_). One common feature of AHR–PINs with D_max_ values greater than 1 was that the majority of M-nodes were interconnected in a single large network with no breaks (Figure 2A–2C). For convenience, we refer to this single large network simply as the AHR–PIN in following discussions. When D_max_ was set at low stringency (D_max_ ≥ 3), the representation of M-nodes in AHR–PIN was high. For example, at D_max_ = 3, 46 of 54 M-nodes were included. However, AHR–PINs resulting from these inclusive, yet low-stringency conditions exhibited high complexity, which made it impossible to assess the interactions visually (Figure 2A and 2B). When D_max_ was set at higher stringency (D_max_ = 2), the resultant AHR–PIN now comprised 34 closely interconnected M-nodes and was much easier to visualize (Figure 2C; Table S4). Further simplification of the AHR–PIN with D_max_ = 1 was of little utility because it resulted in a large proportion of isolated M-nodes, with the largest cluster containing only three M-nodes (Figure 2D).

### The AHR–PIN Is Distinct from Random PINs

To examine the statistical significance of the AHR–PINs, we tested whether they could have been generated by random chance. If the AHR–PIN represents a valid description of the AHR pathway, it should comprise significantly more interconnected M-nodes than would be interconnected by random chance. To test this idea, a Monte Carlo simulation was conducted by generating 5,000 random PINs at each D_max_ setting. Each of these test PINs was constructed based on 54 mock M-nodes randomly selected from genes contained in the entire deletion set. To estimate the statistical significance of the AHR–PIN, the random graph was defined as the null distribution, and the *p* value for the AHR–PIN at each D_max_ was calculated from the fraction of trials with a higher number of interconnected M-nodes (Figure 2E–2H). The AHR–PIN at D_max_ = 1 was not statistically significant compared to those generated at random chance (*p* < 0.08; Figure 2H). However, at D_max_ = 2, D_max_ = 3, and D_max_ = 4, the number of interconnected M-nodes in the AHR–PIN was significantly larger than that of random PINs (*p* < 10^–4^, 10^–3^, and 3 × 10^–3^, respectively; Figure 2E–2G). These observations were consistent with the idea that AHR–PINs at these settings provide a biologically meaningful description of AHR signaling.

For further exploration, we chose to focus on the network with the greatest statistical significance, i.e., the PIN generated at D_max_ = 2. In this AHR–PIN, 63.0% of the M-nodes (34/54) are interconnected, while in corresponding random PINs with mock M-nodes, this number drops to 18.5% (10/54). Although the AHR–PINs at D_max_ = 3 and D_max_ = 4 also exhibited statistically significant differences from random PINs, these AHR–PINs were not considered further for two reasons. First, these networks were visually complex and could not be simply annotated in two dimensions. Second, the ratios of interconnected M-nodes in these AHR–PINs to those of random PINs were quite low (1.3 and 1.2 for D_max_ = 3 and D_max_ = 4, respectively). This observation suggests a much greater potential for displaying false positive interactions at these settings as compared to the AHR–PIN at D_max_ = 2, where this ratio was 3.4 (34/10).

### Modular Organization of AHR–PIN as Revealed by Network Clustering

Our next objective was to use the PIN to enumerate and define steps in AHR signaling. It has been suggested that PINs exhibit a modular nature, with each module comprising highly interconnected proteins of related cellular functions (Hartwell et al. 1999; Schwikowski et al. 2000). Our hypothesis was that functional modules in the AHR–PIN would correspond to discrete steps in the mechanism of signaling. To test this idea, we attempted to define the functional modules using a number of computational and experimental annotation approaches.

**Figure 3:**
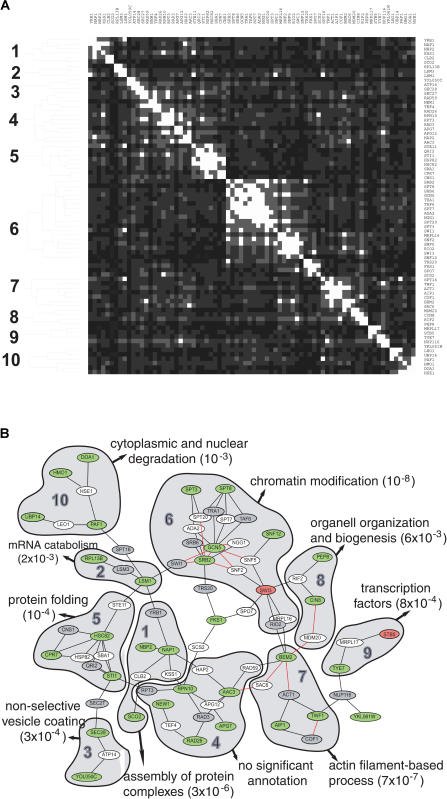
Functional Modules Identified by Network Clustering (A) Network clustering of AHR–PIN. Protein nodes in the AHR–PIN (D_max_ = 2) were clustered by a hierarchical clustering algorithm. A tree-depth threshold was set to delimit cluster boundaries (Rives and Galitski 2003). Clusters with at least two M-nodes are shown. See text for details. (B) Overlay of the network clusters on the AHR–PIN. The ten network clusters correspond to ten local areas in the AHR–PIN. Each network cluster (local area) is labeled with its significant functional enrichment as calculated using the FunSpec program (Robinson et al. 2002). **Color scheme.** Nodes: modifier deletions that incurred down- and up-regulation of AHR signaling are marked in green and red, respectively. For intervening nodes, essential genes are marked in gray and nonessential genes in white. Links: physical interactions are labeled in black and genetic interactions in red. If both interactions are available for a given link, only the physical interaction is shown. This color scheme is also applied to Figures 4–7.

As a strictly computational approach, we attempted to identify the functional modules in the AHR–PIN by a network-clustering method (Rives and Galitski 2003). In brief, an all-pairs-shortest-path distance matrix was generated for every pair of nodes within the AHR–PIN (D_max_ = 2). Each distance (*d*) in the matrix refers to the length of the shortest path between a pair of nodes in the full network space of yeast genomic PIN and was transformed into an “association” value (*1/d^2^*). The resultant pairwise association matrix was used to identify network clusters in the AHR–PIN by a hierarchical average-linkage clustering algorithm (Eisen et al. 1998; Rives and Galitski 2003). The cluster boundaries were delimited by using a similar “tree-depth threshold” that was set low enough to separate the largest cluster from others (Figure 3A) (Rives and Galitski 2003). If we define a network cluster to include at least two M-nodes, ten such clusters can be identified (Figure 3A). Consistent with the modular PIN hypothesis, we found that these clusters overlapped with ten local areas (modules) in the AHR–PIN, with each module comprised of two to six M-nodes (Figure 3B).

In an effort to define the function of these proposed network modules, we asked whether each individual module could be best described by a particular annotation. A module is considered to be enriched for a given annotation if the number of components known to have that function within the module exceeds the number that could be expected from random chance. It has been proposed that the degree of enrichment for a given annotation can be measured by its hypergeometric distribution (Tavazoie et al. 1999). Using this approach, we calculated the annotation enrichment for each of the ten protein modules in the AHR–PIN with the FunSpec program (Table S5) (Robinson et al. 2002). As shown in Figure 3B, it was found that the AHR–PIN is organized by protein modules that perform distinct cellular functions (e.g., protein folding and chromatin modification).

### Functional Modules as Revealed by Their Influence on Different AHR Domains

**Figure 4:**
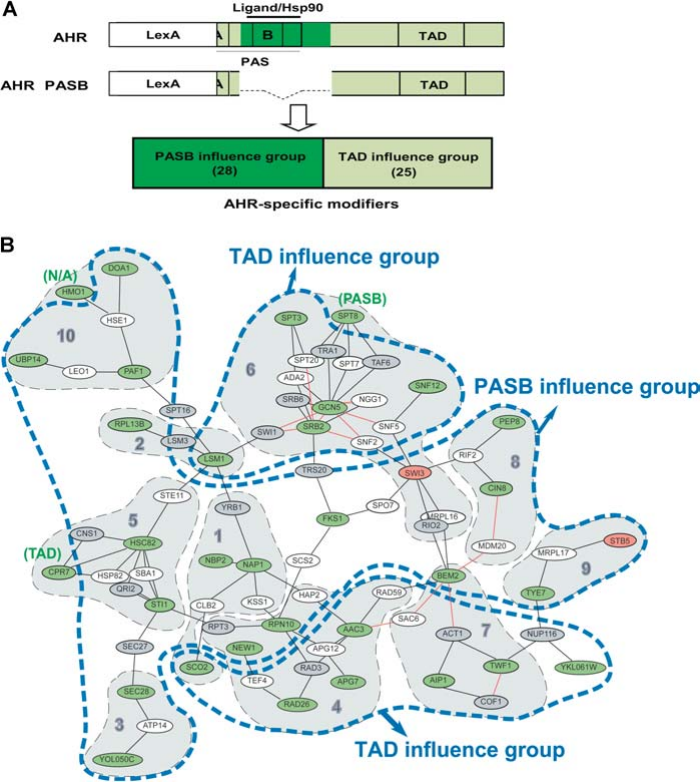
Functional Modules Identified by the “Domain Influence” (A) Identification of domain influencing groups. The effects of modifier deletions on the signaling of AHR and AHRΔPASB were compared in parallel. It was found that 28 modifiers were required for the function of the PASB domain (i.e., their deletions affected the AHR, but not the AHRΔPASB). The other 25 modifiers were found to be required for the shared TAD region (i.e., their deletions affected the signaling of both AHR and AHRΔPASB). (B) Overlay of the “domain influence” layer (blue boundary) and the network-clustering layer (shadowed) on the AHR–PIN. The PASB influence group corresponds to a central region in the AHR–PIN. The TAD influence group corresponds to two peripheral areas. Occasional outlier nodes are marked with their corresponding module names.

In an effort to test the predicted modules and define how they influence AHR signaling, we annotated the AHR–PIN using a number of independent functional tests. First, we examined whether functional modules could be identified based upon their influence on different domains of the AHR. To this end, we examined the influence of each modifier on the signaling of a partial-deletion mutant, pAHRΔPASB, which contains the AHR's transcriptionally active domain but is missing those domains responsible for ligand binding and Hsp90 interaction (Figure 4A). Of the 53 modifier deletions successfully transformed with the pAHRΔPASB system, we found that 25 deletions affected both the parent AHR and the deletion mutant. This observation indicated that these 25 modifiers had an influence on the shared C-terminal TAD region and not on the PASB domain (Figure 4A). These modifiers were referred to as the “TAD influence group.” The remaining 28 deletions, which required the PASB domain for their effect, were referred to as the “PASB influence group.”

When the AHR–PIN was annotated according to the domain influence of each modifier, it was found that modifiers from the same domain influence group closely interacted in the map. That is, the PASB influence group resided in a single connected region, whereas the TAD influence group occupied two peripheral regions (Figure 4B). Interestingly, the PASB module was found to overlap with the computationally identified clusters 1, 3, 5, 8, 9, and 10. For the two TAD modules, one overlapped with cluster 6, and the other with clusters 4 and 7. This overlap supported both the computational and experimental annotations. For example, the “chromatin modification cluster,” 6, identified and annotated computationally, was found to be associated with the TAD influence group, defined experimentally. Similarly, the “protein folding cluster,” 5, was associated with the PASB domain influence group. The PASB domain is known to interact with the chaperone protein Hsp90, which plays a significant role in the folding of the mammalian AHR (Pongratz et al. 1992; Carver et al. 1994; Whitelaw et al. 1995).

### Functional Modules as Revealed by Their Effect on AHR Pharmacology

**Figure 5:**
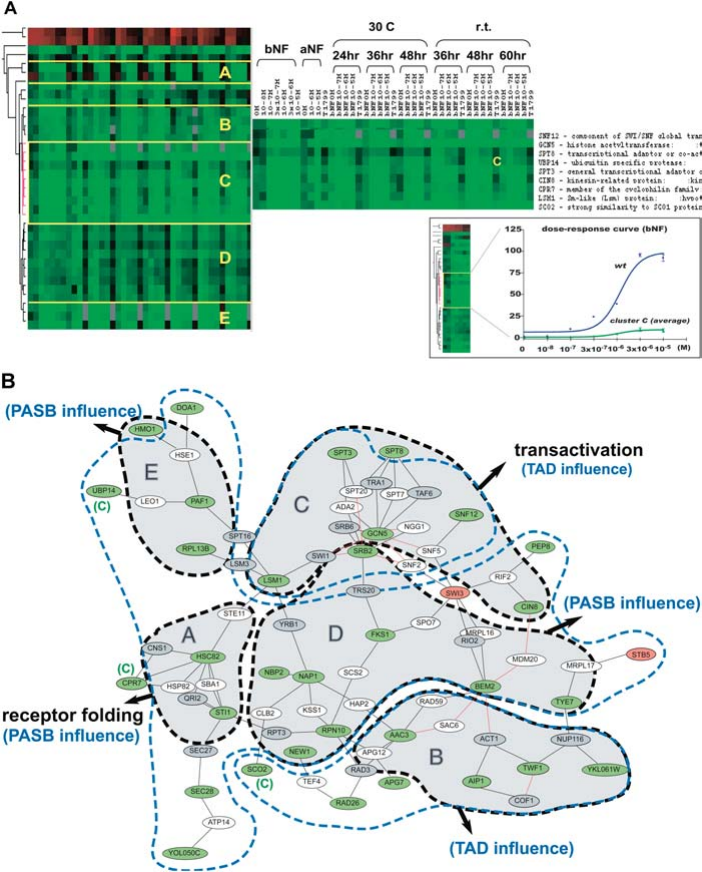
Functional Modules Revealed by Effect on AHR Pharmacology (A) Cluster analysis of the effect of modifier deletion on AHR pharmacology. AHR signaling was examined at various doses, timepoints, and temperatures, and with the two AHR agonists βNF and αNF. The influence of modifier deletion on the dose-response of the AHR was analyzed by a hierarchical clustering algorithm. Rows in the clustering diagram represent modifier deletions. Columns correspond to experimental conditions. Green and red indicate down- and up-regulated AHR signaling, respectively. Color brightness is proportional to fold change. Black indicates *wt* signaling. Sparse gray boxes represent missing datapoints. (Insert) Diagram of corresponding dose-response curves of the *wt* strain and the average of cluster C. (B) Overlay of the “pharmacology clustering” layer (shadowed, black boundary) and “domain influence” layer (blue boundary) on the AHR–PIN. The major pharmacology clusters are coincident with five local areas in the AHR–PIN. In addition, clusters A, D, and E correspond to the PASB influence module, and clusters B and C correspond to the TAD influence module. Functional annotations determined by pharmacology clustering are indicated in black, and those derived from domain influencing are indicated in blue. Occasional outlier nodes are marked with their corresponding module designation. See the legend of Figure 3 for the color scheme of the nodes and links.

To further annotate the AHR–PIN, each of the 54 modifiers was tested for its influence on AHR signaling (pAHR system) at various agonist concentrations, times, and temperatures, as well as after exposure to two distinct AHR agonists, α-naphthoflavone (αNF) and β-naphthoflavone (βNF). The relationship between each modifier and signaling was then examined using a hierarchical average-linkage clustering algorithm (Eisen et al. 1998) (Figure 5A). It was found that the five major clusters corresponded to five closely intraconnected local areas in the map, designated A, B, C, D, and E (Figure 5B). Among them, modules A and C exhibited significant functional enrichment of protein folding and transcriptional control, respectively (data not shown). When the clustering result was overlaid upon the previous maps, it was found that modules A, D, and E corresponded to the PASB influence module, and modules B and C corresponded to the TAD influence module (Figure 5B).

### Functional Modules as Revealed by Their Influence on AHR Localization

**Figure 6:**
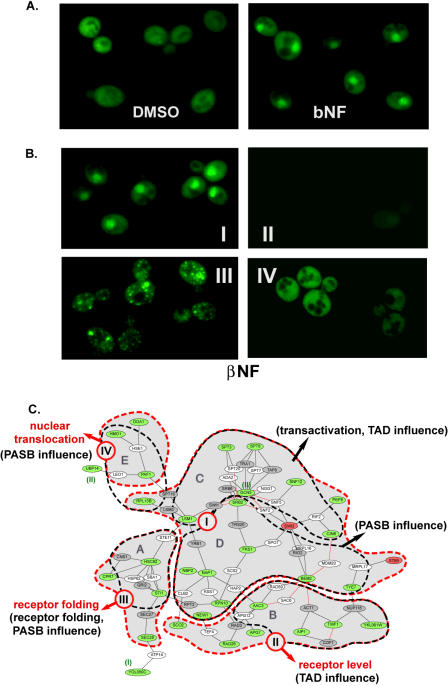
Functional Modules Identified by the “Localization Influence” (A) The AHR–GFP fusion protein translocates to nucleus in the presence of agonist βNF. Nucleus position in the cell was confirmed by DAPI staining (data not shown). Dimethyl sulfoxide (DMSO) is a vehicle control for βNF. (B) Classification of modifier deletion strains according to AHR–GFP phenotype (with βNF). Group I displays *wt* phenotype. Group II contains decreased level of receptor protein. Group III contains aggregated misfolded receptor. Group IV displays the AHR that is not capable of translocating to the nucleus. (C) Overlay of “localization influence” layer (shadowed, red boundary) and the “pharmacology clustering” layer (black boundary) on the AHR–PIN. Group I corresponds to modules C and D. Groups II, III, and IV overlap with modules of B, A, and E, respectively. Functional annotations determined by localization influence are indicated in red, and those derived from pharmacology clustering and domain influencing studies are indicated in black. Occasional outlier nodes are noted with their corresponding module designation. See the legend of Figure 3 for the color scheme of the nodes and links.

Lastly, we examined each modifier's influence on AHR's subcellular localization. This was accomplished using an AHR–GFP fusion protein (pAHRGFP). When the *wt* strain was transformed with the plasmid pAHRGFP, it was found that the fusion protein was evenly distributed in the cell in the absence of AHR agonist. In the presence of the agonist βNF, the AHR–GFP protein translocated to the nucleus (Figure 6A). To examine the influence of each modifier on this translocation process, the pAHRGFP construct was transformed into each of the 54 modifier deletion strains and its localization was examined by fluorescence microscopy in the presence of agonist. Four localization phenotypes were identified (Figure 6B). About 50% of the deletion strains exhibited AHR translocation similar to that observed in the *wt* strain (group I). Approximately 30% of the strains were found to contain a marked reduction in the level of AHR protein in the cell (group II). Approximately 10% of the deletion strains displayed receptor aggregates in the cell (group III). The final 10% of the deletion strains displayed a normal level of AHR protein, but the receptor failed to translocate into the nucleus in the presence of agonist (group IV). When overlaid with the previously determined experimental layers, group I was found to overlap with the modules of C and D, and groups II, III, and IV corresponded to modules B, A, and E, respectively (Figure 6C). According to this overlap, module B can be further described as being associated with the regulation of receptor level in the cell, and module E is associated with the regulation of nuclear translocation of the AHR (Figure 6C).

## Discussion

### Modifier Identification

Our initial objective was to identify the number of loci that are required for AHR signal transduction. In this regard, our high-throughput deletion screen identified 52 novel and two known AHR modifiers. Although this is a surprisingly large number of modifiers for the function of a single protein, it is probably an underestimate since the deletion screen cannot identify modifiers that are encoded by essential genes. Moreover, our criteria of including only strong modifiers (influence of 4-fold compared to control) may have caused us to miss some important modifiers of this pathway. Nevertheless, the number of AHR modifier loci reported here is approximately 10-fold greater than what has been reported using mammalian cell culture and animal models (Schmidt and Bradfield 1996; Whitlock 1999).

Once we identified these AHR modifiers in yeast, we sought a way to position and characterize them in the context of the AHR pathway. Given the idea that PINs can be used to portray the cellular workings, we attempted to use our deletion data to generate and annotate an AHR–PIN (Hartwell et al. 1999; Schwikowski et al. 2000; Ge et al. 2001; Ideker et al. 2001; Tong et al. 2002). To construct the AHR–PIN, the yeast genomic PIN was decomposed by extracting those nodes/links relevant to AHR modifiers. To test the utility of the resultant AHR–PIN, a series of Monte Carlo simulations were carried out. It was demonstrated that when D_max_ was set at 2, 3, or 4, the resultant AHR–PIN was of a complexity that could not have resulted from random chance. Furthermore, the comparison of various simulations at different D_max_ settings guided us to select the linking parameter at D_max_ = 2. This setting of intervening links resulted in the highest level of statistical significance, displayed the lowest potential for false positive interactions, and decreased the map's visual complexity to a level that was readily understood in a two-dimensional map.

### The Modular Structure of AHR–PIN Reveals Five Discrete Steps in Signaling

Our analysis of the AHR–PIN revealed an underlying modular structure. That is, there are areas in the AHR–PIN that display high interconnectedness of nodes, and these regions represent functionally related modifiers. The modularity of AHR–PIN was revealed by both computational and functional tests. In our initial computational approach, a total of ten clusters were identified, and the functional enrichment of each cluster was calculated by hypergeometric distribution (Tavazoie et al. 1999; Robinson et al. 2002).

**Figure 7:**
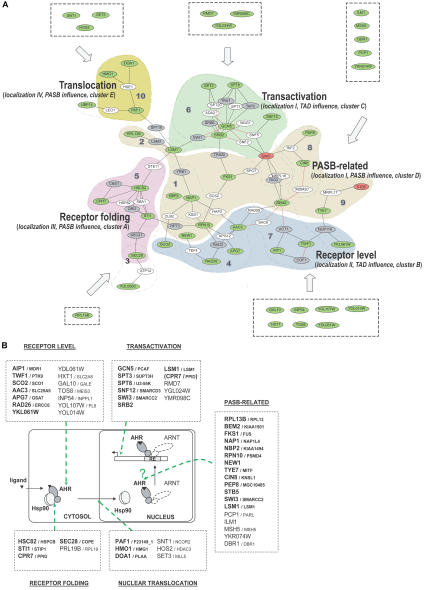
Regulatory Network of AHR Signaling (A) The summary map of AHR–PIN. Functional modules were determined by the overlapped annotations from three experimental layers (domain influence, pharmacology clustering, and localization influence) as well as from network clustering. For each functional module, the main “stacking pattern” of experimental layers is noted in italics. Modifiers initially left outside the single large cluster of the AHR–PIN were assigned to corresponding functional modules by sharing the similar stacking pattern where applicable. See the legend of Figure 3 for the color scheme of the nodes and links. (B) An expanded model of AHR signaling. The AHR signaling pathway is regulated by at least five functional modules that are involved in the control of receptor folding, nuclear translocation, transcriptional activation, receptor level, and a PASB-related nuclear event. Within each functional module, modifers intially enclosed in the single large cluster of the AHR–PIN are highlighted in bold. Known human homologs of the modifiers are noted at the side with a smaller font (Costanzo et al. 2001) . ARNT is dimmed because modifiers were identified in this study from an “ARNT-free” chimeric AHR system. See text for details.

Although the computational approaches of module identification and annotation were useful in hypothesis generation, they did not provide a direct description of AHR signaling. Therefore, we set out to annotate the AHR–PIN with a number of functional tests. In our first annotation experiment (“domain influence”), we found that the AHR–PIN could be divided into three discrete functional modules (i.e., one module that influenced the PASB domain and two modules that influenced the C-terminal domain we referred to as TAD). Additionally, each of these modules was found to overlap with one to several network clusters (see Figure 4). This tight overlay of functional data with highly interconnected regions in the AHR–PIN also held true when we applied annotations for pharmacological clustering and subcellular localization studies (see Figures 5 and 6). Given the overlay of these annotations derived from both functional and computational tests, we conclude that the AHR–PIN provides a biologically meaningful representation of the regulatory network of AHR signaling (Figure 7A). Moreover, based upon the combined annotations for each individual module, we propose that AHR signal transduction is regulated at five discrete steps: (1) receptor folding, (2) receptor translocation, (3) receptor transcriptional activation, (4) receptor level, and (5) a previously undescribed signaling event related to the PASB domain (Figure 7B).

### The AHR Folding Module

A module that regulates AHR folding was identified by the known activities of its constituents, as well as the appearance of receptor aggregates when these modifiers were absent (see Figure 6B, group III). Given that AHR folding has been well studied over the past 15 years, examination of this module provided insight into the fidelity of our screen and the transference of our observations to the mammalian system. For example, two known modifiers were identified by our high-throughput screen: Hsc82p (homolog of human Hsp90) and Cpr7p (homolog of human Cyp40) (Pongratz et al. 1992; Miller 2002). In addition, we identified a previously unknown player in the AHR folding pathway, the chaperone protein Sti1p (homolog of human p60/HOP). Sti1p/p60 has been shown to be an essential component of the glucocorticoid receptor signaling pathway, where it is required to form an Hsp90 chaperone complex (Chang et al. 1997; Dittmar and Pratt 1997). By analogy, we propose that Sti1p/p60 is involved in the formation of an Hsp90·cochaperone complex that is essential for the proper folding of the AHR. Finally, our analysis of this module suggests that a number of proteins not known to be chaperones are involved in receptor folding. These proteins include Sec28p and possibly Rpl19b.

The AHR folding module can also be used to explain the existence of I-nodes within a functional module. Given their “linker” position and the observation that they often share similar annotated function with their neighboring M-nodes (data not shown), it is a logical prediction that I-nodes play a role in AHR signaling that is functionally similar to their modifier neighbors. We propose that I-nodes most commonly arise as the result of their essential gene nature (gray nodes in the figure; nontestable in the deletion screen) or because they represent a redundant gene product (white nodes in the figures). We offer two examples that support this idea. First, one essential gene I-node in the folding module, Cns1p, has recently been reported to be involved in AHR signaling (Miller 2002). Second, the possibility that white nodes may often result from redundancy is supported by what we know about Hsp90. The Hsc82p and Hsp82p proteins are yeast orthologs of human Hsp90, a well-studied chaperone required for proper AHR folding (Pongratz et al. 1992; Carver et al. 1994; Whitelaw et al. 1995). Under normal growth conditions, Hsp82p and Hsc82p account for 7% and 93% of the total “Hsp90 level,” respectively (Borkovich et al. 1989). Thus, it is not surprising that Hsp82p was not identified as a modifier, since its deletion would have had little effect on the total Hsp90 level in the cell (Figure 7A). Finally, white I-nodes can also arise from weak modifiers that influenced AHR signaling by less than 4-fold, e.g., Sba1p (ortholog of human AHR modifier p23) (Kazlauskas et al. 1999). In this regard, although a choice of 4-fold was somewhat arbitrary, we found that lowering the cutoff greatly increased the network complexity without enhancing the statistical significance of the AHR–PIN (as compared with random PINs; data not shown).

### The AHR Employs a Multistep Transcriptional Mechanism

The composition of the transcriptional activation module suggests that the AHR activates target genes via the coordination of histone acetylation, ATP-dependent chromatin remodeling, and direct recruitment of basal RNA polymerase II transcriptional apparatus (see Figure 7). We base this idea on the observation that this functional module is composed of components of the histone acetyltransferase SAGA complex (homolog of the mammalian PCAF complex)—Gcn5p, Spt3p, and Spt8p; components of the SWI/SNF chromatin-remodeling complex—Snf12p and Swi3p; and a subunit of the Srb–mediator complex—Srb2p (Grant et al. 1998; Myers et al. 1998; Peterson et al. 1998). This interdependent requirement of three distinct classes of transcriptionally relevant proteins is consistent with observations from mammalian cells, where the involvement of both HAT and SWI/SNF coactivators in AHR signaling has been reported, as has the direct interaction of the AHR with basal transcriptional factors TBP, TFIIF, and TFIIB (Rowlands et al. 1996; Kobayashi et al. 1997; Swanson and Yang 1998; Beischlag et al. 2002; Wang and Hankinson 2002). These collective data support the idea that AHR transactivation is mediated by a multicomponent, synergistic process.

### Nuclear Translocation of the AHR

Our network analysis has also identified a functional module that regulates the ligand-dependent translocation of the AHR (see Figure 7). This nuclear translocation module appears to be associated with the PASB domain, which is known to play roles in both ligand binding and interaction with chaperones (see Figure 4A). This observation is consistent with the idea that ligand exposure releases the AHR from the cytosolic chaperone anchors (Kazlauskas et al. 2001; Petrulis et al. 2003). Although the mechanism for this translocation event remains unclear, it is interesting to note that the “translocation module” overlaps with a protein degradation cluster, cluster 10 (see Figure 7A). This observation suggests that the underlying control of subcellular localization of the AHR might be related to the selective degradation of certain tethering factors by ubiquitination, possibly mediated by Doa1p and other members in this module (Hochstrasser and Varshavsky 1990).

### Regulation of AHR Expression

A module that regulates the amount of receptor protein was also identified in our AHR–PIN (see Figure 7). This module is associated with the C-terminal domain of the AHR (see Figure 4A). Although we have commonly referred to this region as the TAD domain, these data suggest that other functions are also encoded here. We base this assessment on two observations. First, members of this module are not known to play direct roles in transcription (see Table S4). Second, this module influences receptor level in a manner that is upstream of the AHR's activity as a transcription factor. Our interpretation of this module is that these modifiers are associated with a domain that is proximal to or overlaps with the receptor's TAD and that this domain plays a role in the regulation of receptor level (see Figure 4A). At the present time it is not clear whether this module influences the AHR at its mRNA or protein level.

### A Novel Step Defined by the PASB Module

A novel PASB-dependent step in AHR signaling appears to have been revealed by this network analysis (see Figure 7, PASB-related module). Given that corresponding deletions of this PASB-related module did not impair the receptor's nuclear translocation (see Figure 6, group I), we conclude that this module must influence either a downstream nuclear event or some cytosolic event that is not revealed until the receptor is within the nuclear compartment. On the other hand, this module did not appear to be involved in the final transactivation step, as it was distinct from the transactivation module according to our functional annotations (see Figures 4 and 5). Taken in sum, there must exist a PASB-dependent event that is posttranslocation and pretransactivation. Such an event could be related to the receptor's dimerization, DNA binding, or an as-yet-undefined nuclear event, such as the unfolding of a transcriptionally active domain (Sun et al. 1997; Heid et al. 2000). Interestingly, the existence of this PAS-related signaling is consistent with the previous observation that the DNA binding ability of the AHR can be impaired by a point mutation within its PAS domain (Sun et al. 1997). Lastly, the fact that this PASB-related module overlaps with multiple network clusters (1, 2, 8, 9) suggests a cooperative mechanism that involves more than one cellular function (see Figure 7A).

### Conclusion

We began this study with the objective of defining the AHR signal transduction pathway in a manner that would allow us to quantify the number of loci and enumerate the steps involved in signaling. By integrating our deletion screen with the PIN framework and through subsequent computational and experimental annotations, we were able to identify modifier modules that regulate five distinct AHR signaling steps. In this regard, we found that the integration of multiple annotation approaches is vital for the reconstruction of the final picture by connecting and cross-validating individual information pieces. As interaction datasets become more fully developed and annotated, such a map will steadily improve and provide more accurate description of AHR signaling. Lastly, the systematic strategy that we developed in this work should be readily applicable to the study of most mammalian proteins to reconstruct corresponding modifier networks that regulate their signaling.

## Materials and Methods

### 

#### Strains and plasmids

A set of deletion derivatives of S. cerevisiae strain BY4742 *(MATα, his3Δ1, leu2Δ0, lys2Δ0, ura3Δ0)* was used in this study. This deletion set was obtained from Research Genetics (now a part of Invitrogen, Carlsbad, California, United States) in a 96-well arrayed format. The plasmid pCEN-AHR (PL1605) was constructed by replacing the *TRP1* autotrophic marker of PL883 (Hogenesch 1999) with a *HIS3* marker using a “marker swap” method (Cross 1997). This CEN-based plasmid contains the LexA–AHR chimera cDNA (LexA-AHRNΔ166) under the control of an alcohol dehydrogenase I (*ADH1*) promoter. LexA-AHRNΔ166 is a chimeric AHR, with its amino acid residues 1–166 replaced by residues 1–202 of bacterial repressor LexA, and is referred to in the Results section simply as “AHR” for convenience. The reporter plasmid pSH18–34 (PL623) (Clontech, Palo Alto, California, United States) is a 2μ-based, *URA3*-selectable vector that contains the bacterial *LacZ* gene, as a reporter, under the control of eight LexA-binding sites. The plasmid pEG202 (Clontech, Palo Alto, California, United States) is a 2μ-based, *HIS3*-selectable plasmid containing the LexA_1–202_ sequence under the control of the *ADH1* promoter. The plasmid pAHR (PL700) has been described previously (Carver 1996). This plasmid contains the AHRNΔ166 sequence inserted into the EcoRI site of pEG202. The pGal4TAD control plasmid (PL1573) (Display Systems Biotech, now NeuroSearch A/S, Ballerup, Denmark) contains the transcription activation domain of yeast *GAL4* inserted into the EcoRI site of pEG202. The control plasmid pAHRΔPASB (PL1799) is the same as pAHR except for the removal of the C-terminal half of the PAS domain. This pAHRΔPASB plasmid was constructed by subcloning the EcoRI fragment of PL248 (Carver et al. 1998) into the EcoRI site of pEG202. The plasmid pAHRGFP (PL1890) was constructed as follows: the GFPS65T cassette (Heim et al. 1995) was amplified by PCR from pRSETBGFPS65T (PL1803) (a generous gift from Dr. Catherine Fox, University of Wisconsin–Madison) using primers OL4125 (5′-ACAGCTCTGAAATTCCAGGTTCTCAGGCATTCCTAAGCAAGGTGCAGAGTGGTCGGGATCTGTACGACGAT-3′) and OL4126 (5′-TTAGCTTGGCTGCAGGTCGACTCGAGCGGCCGCCATGGTCGACGGATCCCACCAGCTGCAGATCTCGAGCT-3′). The amplicon was cloned into the DraIII*-*digested pAHR by a gap repair method (Lundblad and Zhou 1997). The resulting plasmid was designated PL1855. The coding sequence for amino acids 1–166 of the AHR was amplified by PCR from PL65 (Dolwick et al. 1993) using primers OL4176 (5′-GCTATACCAAGCATACAATCAACTCCAAGCTTGAATTAATTCCGGGCGGAATGAGCAGCGGCGCCAACAT-3′) and OL4177 (5′-CCTTGTGCAGAGTCTGGGTTTAGAGCCCAGTGAAGCTGGCGCTGGAATTCCGCCCGGTCTTCTGTATGGA-3′). The amplicon was cloned into the PmeI/MluI-digested PL1855 by gap repair. The resultant plasmid was designated pAHRGFP (PL1890).

#### High throughput yeast deletion array transformation

A high-throughput protocol was developed for 96-well transformation based on work previously described (Chen et al. 1992). Unless otherwise noted, all steps were performed with a Hydra 96-channel dispenser (Robbins Scientific, Sunnyvale, California, United States) and a vortex mixer with a microwell plate adaptor (#12-812 and #12-812C, Fisher Scientific, Hampton, New Hampshire, United States). Deletion strains were stored in a stack of 96-well plates (–80 °C). For transformation, each stock plate was thawed and cells were gently resuspended by vortexing. About 0.5 μl of each strain culture was transferred to a 96-well round bottom target plate (Costar #3795, Corning Inc., Acton, Massachusetts, United States) containing 96 μl per well of yeast extract–peptone–dextrose (YPD) medium plus G418 (200 mg/l). This transfer was accomplished with a 96-pin disposable replicator (GenomeSystems, now Incyte Genomics, Palo Alto, California, United States). The inoculum was incubated at 30°C without shaking until the OD_600_ absorbance of individual wells reached 0.2–0.7 (approximately 18 h). The OD_600_ was measured using a SpectraMax 250 microplate reader (Molecular Devices, Sunnyvale, California, United States). Cells were then subjected to centrifugation at 3,500 rpm for 8 min, and the supernatant was decanted. The 96-well plates were placed upside-down on a stack of paper towels for 10 min to drain residual medium. For transformation, each plate was vortexed at maximal speed for 15 s before dispensing 22 μl of DNA in “OneStep” buffer (V_1M LiAc_:V_50% PEG 3350 _= 1:4, with BME added to 0.77% V before use) into each well. To make the DNA in “OneStep” buffer, one volume of DNA (5 μg/μl ssDNA, 0.1 μg/μl each plasmid DNA) was mixed vigorously by vortexing with ten volumes of “OneStep” buffer. After DNA was dispensed, the plate was quickly vortexed again at maximal speed for 10 s to resuspend the cells, followed by incubation at 45°C for 40 min. After this “heat shock” step, 5 μl of the transformation mix from each well was inoculated into a fresh 96-well flat-bottomed plate containing 96 μl per well of dropout medium without Trp, Ura, and His (dropout minus TUH medium) plus G418. The inoculum was gently mixed by vortexing and incubated at 30°C for about 4 d until transformants grew out.

#### The 384-well fluorescence assay for *LacZ* reporter

To perform the *LacZ* reporter assay, transformants from the 96-well plates were rearrayed into 384-well stock plates containing 30 μl per well of dropout minus TUH medium. The inoculum was incubated at 30°C for 2–3 d to allow cell growth. For the *LacZ* reporter assay at each agonist concentration, 0.5 μl of cell culture was transferred from the 384-well stock plate (30°C) into a clear-bottomed/black-walled 384-well assay plate (Falcon #353962, Becton Dickinson, Franklin Lakes, New Jersey, United States) using a disposable 384-pin replicator (GenomeSystems/Incyte Genomics). In the 384-well assay plate, each well contained 18 μl of dropout minus TUH medium (diluted 1:4 in water) plus agonist at the tested concentration. The plates were then incubated at 30°C for 48 h to allow all strains to reach stationary phase. Cell growth was monitored by measuring the OD_600_ of each well using a SpectraMax Plus^384^ microplate reader (Molecular Devices). To initiate the fluorescence assay, 18 μl of lysis/assay buffer was added to each well. Lysis/assay buffer contained a mixture of CUG substrate (#F-2905, Molecular Probes, Eugene, Oregon, United States), 10% SDS, 1 M NaPO_4_, and 25× TAE in the ratio of 1:1.4:350:17.5. For assays with pCEN-AHR transformants, no TAE was required. Plates were vortexed at medium speed for 1 min and left at room temperature for 20 min. The reaction was stopped by dispensing 6.5 μl of 25× TAE to each well and vortexing at medium speed for 1 min. The fluorescence emission of each well was detected using a Wallac “VICTOR V” microplate reader (Perkin-Elmer, Boston, Massachusetts, United States). The fluorescence reading was normalized to the corresponding OD_600_ value to obtain the LacZ reporter activity of each deletion strain.

#### In vivo microscopic analysis of AHR–GFP localization

Selected deletion strains were transformed with the plasmid pAHRGFP. Transformants were incubated in a 96-well microtiter plate containing 100 μl per well of dropout minus TH medium at room temperature. Given that we have observed that small temperature shifts can affect AHR's localization, we found it more convenient to both grow and examine cells at the same temperature. For some samples, assays were repeated at 30°C using a heating chamber attached to the microscope. Such results were found to be comparable to those obtained at room temperature. For strains that reached early log phase, 0.5 μl of culture was mounted on a glass slide, and the AHR–GFP subcellular localization was examined using a Zeiss (Oberkochen, Germany) Axiovert 200M microscope (α Plan-FLUAR 100× objective). Images were captured using an AxioCam HR digital microscope camera (Zeiss). To stain the nucleus in living cells, 4,6-diamidino-2-phenylindole (DAPI) was added to the dropout minus TH medium to a final concentration of 5 μg/ml.

#### Modifier identification and network analysis

To identify deletions that modify AHR signaling, the *LacZ* reporter activity of each deletion strain was compared to the average of *wt* BY4742 strain controls included in the same plate, and the fold change was obtained and log_2_ transformed. These data-processing steps, as well as subsequent modifier selection, were performed automatically using Perl scripts written “in house.” In brief, for the primary screen involving 4,507 deletion strains with low-copy pCEN-AHR system, a stringent cutoff of 4-fold change over *wt* control was chosen for selecting a pool of most significant AHR signaling mutants. This cutoff corresponds to a *p* value of less than 10^–6^ at all six assessed concentrations (null distribution: *wt* control). The initial positives were subject to validation and characterization in secondary screens with high-copy pAHR and control systems. The cutoffs for control pathways pGal4TAD and pAHRΔPASB in the secondary screens were chosen at 2-fold change over *wt* control, which corresponds to *p* values of 3.3 × 10^–2^ and 5.6 × 10^–4^ (null distribution: *wt* control), respectively.

For PIN construction, the main physical interaction table was downloaded from the DIP database (http://dip.doe-mbi.ucla.edu) and the genetic interaction table from the MIPS database (http://mips.gsf.de/proj/yeast/). Perl scripts, written “in house,” were used to search the combined physical and genetic interaction database and identify all valid paths (less than or equal to D_max_) that linked each pair of modifiers. The network graph was rendered using the Graphviz tool kit (http://www.research.att.com/sw/tools/graphviz/) (Ellson et al. 2004).

Within experimental annotation layers of the AHR–PIN, the region corresponding to each functional module was outlined by a closed line (boundary) drawn manually on the network map. This boundary was delineated to include the maximal number of modifier nodes that are members of the corresponding functional module and the minimal number of modifier nodes that are nonmembers. This boundary was also defined in such a way that all enclosed modifier nodes were interconnected via paths within the enclosed region or through at most one modifier node outside. When defining functional modules in the summary AHR–PIN, the highest weight was given to the results from the localization influence experiments because these results provided the most direct indication of a modifier's effect on AHR signaling, and the lowest weight was given to the pharmacology clustering result because this result was highly sensitive to the choice of clustering algorithm.

## Supporting Information

Table S1 Significant AHR Modifiers This table contains all of the ORFs whose corresponding deletion strains reproducibly displayed a significant change in AHR signaling compared to *wt* BY4742 strain. Also shown are their known gene names, products, gene descriptions, and Gene Ontology (GO) annotations (Ashburner et al. 2000;  Issel-Tarver et al. 2002).
(35 KB XLS).

Table S2 AHR-Specific Modifiers This table contains all of the ORFs that were observed to influence the signaling of the AHR but not the pGal4TAD control. Also shown are their known gene names, products, gene descriptions, and GO annotations (Ashburner et al. 2000; Issel-Tarver et al. 2002).
(27 KB XLS).

Table S3 YPD Annotation of AHR Modifiers This table summarizes the annotation on cellular functions of AHR modifiers. The annotation was derived from the YPD database, as of May 2002 (Costanzo et al. 2001) .
(23 KB XLS).

Table S4 M-Nodes in the AHR–PIN This table contains all of the AHR modifiers that were interconnected in the AHR–PIN (D_max_ = 2). Also shown are their known gene names, products, gene descriptions, and GO annotations (Ashburner et al. 2000; Issel-Tarver et al. 2002).
(24 KB XLS).

Table S5 Functional Enrichment of Network Clusters This table summarizes the functional enrichment of each network cluster as calculated by the hypergeometric distribution of MIPS and GO annotations. For each cluster, the functional enrichment is determined by using M-nodes alone and both M- and I-nodes, respectively. In each case, the annotation that corresponds to the largest number of nodes in the cluster and the smallest *p* value is shown (*k*, number of genes from the query cluster in the given category; *f*, total number of genes in the given category).
(22 KB XLS).

## Abbreviations

*ADH1*: alcohol dehydrogenase I
AHR: aryl hydrocarbon receptor
DAPI: 4,6-diamidino-2-phenylindole
DRE: dioxin responsive enhancer
GO: Gene Ontology
HAT: histone acetyltransferase
αNF: α-naphthoflavone
βNF: β-naphthoflavone
ORF: open reading frame
PAH: polycyclic aromatic hydrocarbon
PAS: Per–Arnt–Sim
PIN: protein interaction network
TAD: transcriptional activation domain
TUH: Trp,Ura
*wt*: wild-type
YPD: yeast extract–peptone–dextrose
